# Infective endocarditis presentations during the COVID-19 pandemic: have they paid an untold toll?

**DOI:** 10.21542/gcsp.2024.11

**Published:** 2024-03-03

**Authors:** Ahmed Elamragy, Ahmad Samir, Ahmed Maher, Hussein Rizk, Marwa Meshaal

**Affiliations:** 1 Kasralainy Medical School, Faculty of Medicine, Cairo University, Cairo, Egypt

## Abstract

**Background**: COVID-19 caused restrictions and re-allocation of medical resources among all healthcare services. During the peak of the pandemic, several unrelated–yet critical–conditions had silently taken their toll. Infective endocarditis (IE), owing to its non-specific clinical presentation, may have been largely mislabeled as COVID-19 in a number of cases.

**Results**: This retrospective observational study reviewed all IE presentations at an IE unit in a university hospital during the peak of COVID-19. Patient characteristics, courses, and outcomes were compared with historical controls from our IE database published before the COVID era. We identified 30 IE cases [Group A] during the COVID-19 peak in our region (June 2021 to June 2022), with a 25% decrease compared to the usual annual rate. This is in contrast to the expected surge during the pandemic. Compared with group B (398 published IE cases from our database), group A had significantly longer symptoms-to-presentation intervals (60 [31–92] *vs.* 28 [14–72] days, *p* = 0.01). Male sex dominated both groups, but group A had significantly less pre-existing structural heart disease. Despite the more liberal use of empirical antibiotics in the COVID-era, group-A had lower rates of culture-negative IE. Compared to group B, group A demonstrated a better response to medical therapy, fewer arterial embolizations, fewer indications for surgery, and fewer overall complications, except for increased acute kidney injury. This can be explained by the abundant use of non-steroidal anti-inflammatory drugs. The data analysis strongly suggests that there might have been a natural selection or selection bias of IE patients with favorable profiles to survive the pandemic to the appropriate diagnosis.

**Conclusions**: The diagnosis of IE and commencing the appropriate workup were significantly undermined during the COVID-19 pandemic. The inexplicable decline in IE referral rate and the favorable outcomes witnessed during the pandemic strongly suggest a referral bias and natural selection of those who survived the pandemic to the appropriate IE diagnosis.

## Background

Coronavirus disease 2019 (COVID-19) has led to serious and major consequences in all aspects of life^1^. It poses a massive burden on healthcare systems worldwide, even the strongest and most developed ones^2^. During the pandemic, particularly with the regional peaking of its waves, there were critical shortages in healthcare personnel, hospital bedding, and medical supplies, which greatly impacted the efficacy and rigorousness of medical practices.

In retrospect, we have realized that the high infectivity and rapid community spread of severe acute respiratory syndrome coronavirus 2 (SARS-CoV-2) has allowed no time to analyze the problem or to plan effectively. Lockdowns, curfews, healthcare resources reallocation, and other similar approaches were proposed as bailout solutions for the pressing situation^3^. During the peaking waves of the pandemic, all aspects of medical practice were substantially undermined, including cancellation of elective visits and procedures, and deferral of all “judged as non-emergency” medical services in order to limit the spread of the virus^4,5^.

Infective endocarditis (IE), although a virulent condition with high case morbidity and fatality rate, often starts with atypical presentation with non-specific constitutional manifestations, and hence requires a low-threshold of suspicion and a high-experience to suspect and diagnose early. The COVID-19 pandemic seems to have had a significant, yet concealed, impact on IE admissions. Several reports have suggested a 7-fold increase in hospital-associated IE (HAIE) incidence during the COVID pandemic^6^, while many others claimed that in many instances, IE diagnosis was substantially delayed or even confused as COVID-19 infection^7^.

In this study, we compared the clinical profile, hospital course, and outcomes of IE patients during the COVID-19 peak with a previously published historic control from the IE database^8^, in a tertiary care institute.

## Methods

### Study design and population

This retrospective observational analytic study was conducted in the IE-specialized unit of the cardiology department of a university hospital. This IE unit and its team serve as a referral center for several hospitals and medical facilities. We retrospectively recruited all patients diagnosed with definite/possible IE between June 2021 to June 2022 [the peak for COVID-19 pandemic in our region] -as the COVID-era patients(Group A). They were contrasted with our previously published IE database^8^, which served as a historical control (Group B: pre-COVID-19 era patients), aiming to explore the potential impacts of the COVID pandemic on IE referrals.

It should be mentioned that the main source of cases to our unit is from referrals of suspected/diagnosed IE cases from other facilities in our network and from complicated cases in other departments (medical or surgical) of our university hospital. This might have led to a referral bias towards more complex cases with higher rates of intra- and extra-cardiac complications, as demonstrated in previously published reports from our group^8–11^.

### Ethics approval

All IE patients approved *via* written informed consent that their anonymized clinical data can be registered into the institutional IE database, permitting that pooled data analysis devoid of any personal identifiers can be used for clinical research and scientific publications. The study protocol and methodology were reviewed and approved by the institutional Research Ethics Committee.

### Diagnostic workup

As part of our local institutional protocol, and in line with guideline recommendations^12^, basic clinical characteristics such as patients’ age, gender, comorbidities, injecting drugs, and other relevant features to IE predisposition(such as structural heart disease, recent hospitalization, or intravenous (IV) drug abuse), were detailed for all patients. The time to consider IE diagnosis was defined as the time (in days) between the onset of symptoms that were likely caused by IE(fever, malaise, body aches, hematuria, vascular, or neurologic events) and the time of suspecting IE and starting its workup(withdrawing blood cultures or performing echocardiography). Although starting IE-specific therapy could be an appealing time-point, it varied according to the patient’s presentation between same-day (after accelerated blood culture withdrawal over 3 h for unstable patients), up to a few days delay for antimicrobial clearance, and conventional blood culture withdrawal regimen (in stable patients with prior empirical antibiotic exposure). Therefore, we resorted to the time of medical consideration of IE diagnosis as a more standardized time point.

Clinical diagnosis of IE was based on the modified Duke criteria and followed the European Society of Cardiology guidelines for the diagnosis and management of IE^12,13^. A comprehensive workup for IE was completed, and we excluded those with rejected IE diagnoses, ensuring that there was an alternative diagnosis to explain the clinical presentation. Therefore, through both periods (pre-COVID-era and the COVID-era), patients with two major, one major and three minor, or five minor criteria according to modified Duke’s definitions were considered definite IE, while those with one major plus one or two minor, or with three or four minor criteria were considered as possible IE cases, and these represented the inclusion criteria for the present study analysis^12^.

Our institutional protocol for IE workup also included blood cultures, serological testing, inflammatory marker assessment, echocardiogram, fundus examination, and imaging for vascular complications, as warranted by the clinical scenario, as detailed in previous publications^8,9^.

Blood cultures comprised three or more sets of cultures (aerobic and anaerobic) withdrawn by direct sampling from a peripheral vein under aseptic conditions. Serological tests included Brucella, Bartonella, Coxiella, and Galactomannan for Aspergillus, while other assays were individualized according to the clinical scenario.

A transthoracic echocardiogram (TTE) was performed for all patients within 24 h of hospital admission. Transesophageal echocardiography (TEE) was also performed for all patients within another 72 h (excluding those with hemodynamic instability and right-sided IE patients with sufficient TTE data and quality). All TTE/TEE views and definitions of vegetations and intracardiac lesions were standardized according to the guidelines of the European Society of Cardiology for the management of endocarditis^12^ and the consensus documents from the European Association of Echocardiography and American Society of Echocardiography^13,14^.

Fever was counted when the measured core body temperature was ≥37.7^^∘^^ C. Structural heart disease was defined as congenital, rheumatological, or inflammatory abnormalities affecting the cardiac valves or great vessels, including aortic coarctation or patent ductus arteriosus. Any prosthetic heart material, surgically or percutaneously implanted, such as occluder devices, plugs, conduits, and patches, are covered by this definition. Rheumatic heart disease was considered when there was presence of any rheumatic valvular features on echocardiographic evaluation^15^, and was supported by a history suggestive of rheumatic fever during childhood or teenage years. Healthcare-associated IE (HAIE) refers to IE episodes likely related to healthcare services in outpatient clinics, dental procedures, intravenous (IV) catheters or IV injections, or those involving invasive or surgical procedures performed in a medical facility^16^. Drug injection-associated IE, or as recently described, persons who inject drugs (PWID), include those with repeated IV drug administration outside a medical facility, either for substance abuse or non-professional administration of clinically indicated medications^17^. Prior IE referred to previous episodes of definite/possible IE diagnosis that warranted extended antimicrobial therapy and/or therapeutic cardiac interventions. According to the guidelines, an IE relapse was defined as a repeat infection caused by the same microorganism, while IE reinfection was diagnosed when it was caused by a different organism^13^.

### Statistical analysis

Data were expressed as frequencies (percentages) for categorical variables. For continuous variables, the normality of distribution was first evaluated using Shapiro–Wilk testing and/or histograms, accordingly, data were expressed as median [25th percentile–75th percentile] or mean ± standard deviation (SD) for non-normal and normal distributions, respectively. Categorical variables were compared between groups using Chi-square or Fisher-Exact test as appropriate, while for continuous variables, independent-samples- *t*-test or Mann–Whitney test were used as mandated by distribution.

## Results

In this observational study, we included 428 patients in two groups: group A consisted of 30 consecutive IE patients who were recruited during the COVID-era, and group B included 398 IE patients from our previously published data^8^.

Overall, 30 IE patients/year represented a drop of 25% compared with the average annual rate of 40 IE referrals to our unit in the later years of our program^8^. The median age(interquartile range [IQR]) of group A *vs.* group B was 37 [28–46] *vs.* 30 [23–39] years, respectively (*p* = 0.01), while male sex was predominant in both groups. The median time from IE-suggestive symptoms-to-consideration of IE diagnosis was significantly longer in group A than in group B (60 [31–92] *vs.* 28 [14–72] days, respectively; *p* = 0.007). Other patient demographics, presentations, and clinical and laboratory profiles were compared between the two groups (Table 1).

**Table 1:** Clinical and laboratory features of the whole study group, and comparative analysis of group A (COVID-19-era) and group B (control). Group A: patients in the COVID-19 era. Group B: a control group of patients in the registry before the COVID-19 era.

**Clinical data**	**Group A**	**Group B**	***P* value**
	**(n: 30)**	**(n: 398)**	
Age (years)	37 [28–46]	30 [23–39]	0.01
Male gender	19 (63.3%)	243 (61.1%)	0.81
Time from IE-symptoms to consideration of diagnosis (days)	60 [31–92]	28 [14–72]	0.007
Fever as a presenting symptom	26 (86.7%)	335 (84.2%)	1.0
Injecting IV drugs	6 (20.0%)	42 (10.6%)	0.13
Structural heart predisposition	14 (46.7%)	255 (64.1%)	0.057
Prosthetic valve	6 (20%)	104 (26.1%)	0.46
Procedures in the last 3 months	3 (10%)	95 (23.9%)	0.08
Prior Endocarditis	3(10%)	15(3.8%)	0.12
Temperature on admission (°C)	39 [38–39]	38 [38–39]	0.003
**Laboratory data**			
Culture-negative IE	15 (50%)	275 (69.1%)	0.031
Hb on admission (g/dl)	8.8 (8.0–11)	9.5 (8.0–11.0)	0.31
Hb lowest level (g/dl)	9.6 (8.4–10.6)	8.9 (7.3–10.5)	0.72
Hb on discharge (g/dl)	9.7 (8.5–11.0)	10.3 (9.0–11.2)	0.07
Cr on admission (mg/dl)	1.0 (0.8–1.1)	0.9 (0.7–1.3)	0.32
Maximum Cr (mg/dl)	1.2 (0.98–1.6)	1.6 (0.96–2.8)	0.15
Cr on discharge (mg/dl)	0.8 (0.8–1.0)	1.0 (0.7–1.4)	0.35
Baseline ESR	115 (82–120)	100 (60-120)	0.12
Final ESR	20 (20–32)	50 (20–90)	0.003
Baseline TLC (/mm^3^ )	12 [9–17]	11 [8–15]	0.33
Final TLC (/mm^3^)	6.3 [4.7–7.0]	7.8 [6–9.9]	0.003

**Notes.**

Cr Creatinine ESR Erythrocyte sedimentation rate Hb Hemoglobin IE Infective endocarditis IV Intravenous TLC Total leucocytic count

Data presented as frequency (percentage) or median [25 th -75 th percentiles] as appropriate.

The frequency of IV drug injection was almost double in group A compared to that in group B, but the difference was not statistically significant (20% *vs.* 10.6%). Compared to group B, group A showed a trend toward lower rates of structural heart disease predisposition (48.3% *vs.* 64.1%, *p* = 0.057) and for undergoing elective medical procedures in the previous 3 months (10% *vs.* 23.9%, *p* = 0.08). There was no difference in sidedness (left- *vs.* right-side valves) of IE in both groups; however, group A had an almost 3-fold higher rate of prior IE than group B (10% *vs.* 3.8%), yet without achieving statistical significance.

### Clinical parameters and hospital course

The disease course and outcomes revealed significantly higher grades of fever on admission and lower rates of culture-negative IE than those in group B. Staphylococcus aureus dominated the recognized pathogens (representing 64%), followed by streptococci (7%) and enterococci (7%) in group A, which is comparable to the 50%, 12%, and 4% rates, respectively, in group B. Compared to group-B, the majority of IE complications were less observed in group-A (arterial embolization, sepsis, heart failure, neurologic events and indication for surgery), except for AKI, which occurred significantly more in group-A compared to group-B, paralleling the abundant and prolonged use of non-steroidal anti-inflammatory drugs. Despite the numerically higher levels of inflammatory markers (ESR and TLC) in group-A patients on-admission, by the end of the hospital course, they showed significantly lower (better) levels compared to group-B. The in-hospital mortality was comparable in both groups (Tables 1 and 2).

**Table 2:** Group A: patients in the COVID-19 era. Group B: a control group of patients in the registry before the COVID-19 era.

**Outcome data**	**Group A**	**Group B**	**P value**
	**(n: 30)**	**(n: 398)**	
Response to antimicrobials	17 (56.7%)	185 (46.5%)	0.28
Planned for surgery	14 (46.7%)	294 (73.9%)	0.001
Heart failure	9 (30%)	148 (37.2%)	0.431
Major arterial embolization	4 (13.3%)	133 (33.4%)	0.023
Neurologic events	3 (10%)	76 (19.1%)	0.216
Severe sepsis	7 (23.3%)	100 (25.1%)	0.827
AKI	12 (40%)	82 (20.6%)	0.013
Mortality	5 (16.7%)	108 (27.1%)	0.21

**Notes.**

AKI acute kidney injury.

Data presented as frequency (percentage).

## Discussion

The impact of the COVID-19 pandemic on IE presentation and outcomes is complicated and puzzling. During the peaking waves of the pandemic, there was a worldwide refrain not to visit medical facilities for fear of catching COVID, leading to considerable delays in the appropriate diagnosis (and thus under-reporting) of several serious medical conditions^18,19^. Also, the substantial restrictions of medical services, and the tendency to attribute any febrile illness and/or constitutional manifestations as COVID-19, are believed to have caused significant underdiagnosis of IE, or misdiagnosis of IE as COVID-19^20,21^.

Conversely, in parallel with thousands of daily COVID-19 hospitalizations, receiving IV lines and parenteral injections, or receiving parenteral antibiotics or antipyretics in non-professional setups (homes/pharmacies), several reports have stated a substantial increase in the incidence of IE, to the extent that it was suggested to count IE as a cardiovascular complication of COVID-19 infection^22^. Others have reported an increase in IE incidence during the COVID-19 pandemic, both community- and hospital-associated, by up to 7-fold^6,23^.

What further complicates the COVID-19 and IE relationship is the interlinkage in the pathogenesis between both conditions. The intense inflammatory storm complicating COVID-19 has been proved to cause vascular endothelial injury, which is certainly valid for blood vessels and cardiac valve endothelium^21^. Some other reports have implicated SARS-CoV-2 in inducing direct cardiac valve damage and acute valve dysfunction^24^. If added to the susceptibility of COVID-19 patients to various systemic infections due to the liberal use of systemic steroids^25^, one can appreciate how the COVID-19 pandemic could have led to a surge in IE incidence.

Despite this intimate relationship and the high susceptibility of COVID-19 patients to IE, the colossal spread of SARS-CoV-2 drew attention to COVID infection and partially blinded physicians to suspect other etiologies of febrile illnesses. Hence, during the COVID-19 peaking waves, the trend was to label any person with fever, body aches, constitutional symptoms and/or shortness of breath as having COVID-19, which left other–potentially fatal–diseases undiagnosed.

IE patients who tested positive for SARS-CoV-2 by polymerase chain reaction (PCR), either for recent recovery or for being asymptomatic carriers of COVID-19, could be another confounding factor. Evidence that individuals can test positive for SARS-CoV-2 by PCR while being completely asymptomatic has been repeatedly confirmed^26^. In such scenarios, all efforts to seek another diagnosis (like ordering blood cultures or echocardiography) were suppressed, making–at least hypothetically–mislabeling IE presentations as COVID during the pandemic very conceivable^20,21,27^. In several reports, when COVID-19 infections were associated with concomitant IE, despite the often delayed recognition of this dilemma, it was impossible to recognize whether COVID-19 preceded and precipitated IE, or it could have been mislabeled from the outset^21,27^.

In this study, we sought to contrast IE presentations during the COVID-19 pandemic (group A) to our previously published IE database prior to the COVID era (group B) to characterize the pandemic’s impact on such conditions. First, compared to the annual average in recent years, there was a 25% reduction in IE referrals/admissions and a significantly longer time from symptom onset to IE diagnosis. Time-to-diagnosis delays may be attributed to the popular refrain from seeking medical facilities; however, the decline in the number strongly suggests an element of under-reporting. This is serious when compared to a naturally expected surge in IE incidence that should reflect excess hospitalizations, liberal use of systemic steroids, and the escalating non-professional drug injecting behavior (antibiotics or antipyretics) in homes^6,21^.

A terrible tale may have unfurled during the year when COVID-19 peaked in our region, with many supportive surrogates collectively making it more likely to be true. The differences observed between the IE referrals during the COVID-19 era compared to the preceding years would strongly suggest a selection bias, or in other words, “natural selection” of IE cases during the pandemic. Although by simple reasoning, it would have been presumed that later presentations, delayed diagnosis, and forced restrictions in medical services during the pandemic would have led to worse outcomes of IE, we witnessed the opposite. The overall favorable profile, including better responses to medical therapy, better normalization of inflammatory parameters, fewer arterial embolizations, fewer heart failures, fewer indications for surgery, and fewer overall major complications in group A compared to group B, are implausible to be simple coincidences. If added to the declining numbers of referrals opposing the expected natural rise and the declining number of culture-negative IE opposing the widespread use of empirical antibiotics, one should suspect that this is not the whole truth. It is conceivable that many IE patients, particularly those with difficulty in identifying pathogens (representing the majority of culture-negative cases) or those with fast deteriorating courses, had pursued their fates as non-recovering cases in COVID-19 isolation units and were not considered IE cases.

In contrast, those with favorable profiles, without underlying heart disease, easy to detect and treat pathogens, or with prior IE that directed both the patient and the physician to consider IE, represented fortunate patients who survived the chaos that struck the healthcare systems when overwhelmed by the pandemic peak. This arguable scenario makes it very likely that during the pandemic peak, many IE patients paid an untold toll.

If there is a lesson that we should learn from this claim, it is necessary to adhere to diagnostic algorithms equally during crises and times of normalcy. It is undisputable that the COVID-19 pandemic paralyzed many health systems worldwide and hopefully we do not see such recurrences. Yet, if it was to see similar crises, we should stick to appropriate diagnostic pathways rather than following a trending diagnosis, or we may silently lose lots of precious lives.

### Study limitations

This study has some limitations. The chaotic nature of the COVID-19 era disrupted the usual pathways of medical services and our referral system was not spared. Although most of the findings observed in this analysis were striking numerically, many of them lacked statistical significance because of the small sample size in group A. However, the small group number itself, added to the other factors, collectively raise suspicion of the scenario that the authors presume, as it is unlikely these are simple coincidences. Another critical limitation was that patients admitted to COVID-19 isolation units, were out of our reach of the IE team, while such data would have complemented the other half of the scenario.

## Conclusions

The COVID-19 pandemic significantly undermined the diagnosis and referral of IE to our tertiary center. During the peak of the pandemic, it is likely there was a natural selection of IE cases with favorable courses who could survive the struggling medical services to the appropriate diagnosis and management, while many others faced their fate as non-recovering COVID-19 infections.

### List of abbreviations

**Table:** 

AKI	Acute kidney injury
COVID-19	Corona virus disease 2019
CIED	Cardiac implantable electronic devices
Cr	Creatinine
CRP	C-reactive protein
ESR	Erythrocyte sedimentation rate
HAIE	Healthcare associated infective endocarditis
Hb	Hemoglobin
IE	Infective endocarditis
IV	Intravenous
PCR	Polymerase chain reaction
PWID	Persons who inject drugs
SARS-CoV-2	Severe acute respiratory syndrome coronavirus 2
TEE	Transesophageal echocardiography
TLC	Total leucocytic count
TTE	Transthoracic echocardiogram
